# Parental physical activity is associated with objectively measured physical activity in young children in a sex-specific manner: the GECKO Drenthe cohort

**DOI:** 10.1186/s12889-018-5883-x

**Published:** 2018-08-20

**Authors:** Silvia I. Brouwer, Leanne K. Küpers, Lotte Kors, Anna Sijtsma, Pieter J. J. Sauer, Carry M. Renders, Eva Corpeleijn

**Affiliations:** 1 Hanze University of Applied Sciences, Institute of Sport Studies, Zernikeplein 17, 9747 AS Groningen, The Netherlands; 2 Department of Epidemiology, University Medical Center Groningen, Hanzeplein 1, 9713 GZ Groningen, The Netherlands; 3 University of Bristol, MRC Integrative Epidemiology Unit, Oakfield House, Oakfield Grove, Clifton, Bristol, BS8 2BN UK; 4 University of Bristol, Population Health Sciences, Oakfield House, Oakfield Grove, Clifton, Bristol, BS8 2BN UK; 5 University Medical Center Groningen, Faculty of Medical Science, Hanzeplein 1, 9713 GZ Groningen, the Netherlands; 6 University Medical Center Groningen, Lifelines, Hanzeplein 1, 9713 GZ Groningen, The Netherlands; 7 Department of Health Sciences, VU University Amsterdam, Faculty of Science, De Boelelaan 1085, 1081 HV Amsterdam, The Netherlands

**Keywords:** Physical activity, Family, Children, Role model

## Abstract

**Background:**

Physical activity (PA) is important in combating childhood obesity. Parents, and thus parental PA, could influence PA in young children. We examined whether the time spent at different intensities of PA and the type of parental PA are associated with the PA of children aged 4–7 years, and whether the associations between child-parent pairs were sex-specific.

**Methods:**

All the participants were recruited from the Groningen Expert Center for Kids with Obesity (GECKO) birth cohort (babies born between 1 April 2006 and 1 April 2007 in Drenthe province, the Netherlands) and were aged 4–7 years during measurement. PA in children was measured using the ActiGraph GT3X (worn at least 3 days, ≥10 h per day). PA in parents was assessed using the validated SQUASH questionnaire.

**Results:**

Of the *N* = 1146 children with valid ActiGraph data and 838 mothers and 814 fathers with valid questionnaire data, 623 child-parent pairs with complete data were analysed. More leisure time PA in mothers was associated with more time spent in moderate-to-vigorous PA (MVPA) in children (Spearman *r* = 0.079, *P* < .05). Maternal PA was significantly related to PA in girls, but not boys. More time spent in maternal vigorous PA, in sports activity, and leisure time PA, were all related to higher MVPA in girls (Spearman *r* = 0.159, *r* = 0.133 and *r* = 0.127 respectively, P_all_ < .05). In fathers, PA levels were predominantly related to PA in sons. High MVPA in fathers was also related to high MVPA in sons (*r* = 0.132, *P* < 0.5). Spending more time in light PA was related to more sedentary time and less time in MVPA in sons.

**Conclusions:**

Higher PA in mothers, for instance in leisure activities, is related to higher PA in daughters, and more active fathers are related to more active sons. To support PA in young children, interventions could focus on the PA of the parent of the same sex as the child. Special attention may be needed for families where the parents have sedentary jobs, as children from these families seem to adopt more sedentary behaviour.

## Background

Overweight and obesity is a growing problem in children. According to the World Health Organization, more than 42 million children aged under five were estimated to be overweight worldwide in 2013 [1], an increase from 4.2% of the overall population in 1990 to 6.7% in 2010. This prevalence is expected to be 9.1% in 2020 [2]. Compared to normal-weight children, overweight or obese children are four times as likely to be overweight in adulthood, resulting in increased healthcare costs [3–5] and an increased risk of developing health problems such as diabetes, heart disease and certain cancers later in life [5, 6].

Overweight and obesity are a consequence of a disturbed energy balance [7]. An important energy balance-related behaviour in addition to diet, is daily physical activity (PA) [8]. A lack of habitual exercise and PA in young children are related to higher body mass index (BMI) [9], greater skinfold thickness [9, 10], greater fat mass [11] and obesity status [12–14]. To prevent future overweight in children, the determinants of their PA levels should be considered by looking at all aspects of their ecological system, including any obesogenic conditions. The ecological system closest to a child is the microsystem, which includes family, peers, school, health services and religious groups [15], with parents as important socializing agents [16]. Parents strongly determine the social and physical environment of their young children [17]. This influence may also provide an important link between the parents’ PA level and their children’s [18]. Parents influence their children’s PA by providing modelling support (being physically active themselves) and social support (praising the child, watching the child participate in PA, engaging in parent–child co-activity, transporting their children to places where they can be active, and parental encouragement) [19, 20].

Numerous studies have examined the relationship between parenting styles or parental support and children’s PA [21–23]. Several studies have focused on the specific relationship between PA levels in parents and the PA levels of their children. One review found little evidence to support the hypothesis that higher PA levels in parents are associated with higher PA levels in children [24]. Another review showed a mixed pattern of associations between the PA levels of parents and those of their children. Six of the studies that were included confirmed an association, while seven studies found a weak or no association [23]. These mixed findings might be due to heterogeneity in study designs with regard to the number and age ranges of participants, geographical location and the methods used to assess PA.

Assessing PA is particularly difficult in young children. Their activity patterns are less structured than the PA habits of adults, and characterized by relatively short bouts of spontaneous, intense PA [25, 26]. This spontaneous behaviour in children is difficult to summarize and report by observation, so questionnaires or parental reports are prone to measurement error [27]. Objective measurements, for example with tri-axial accelerometers, are likely to capture all movements [28, 29]. Accordingly, the use of tri-axial accelerometry to obtain more valid and precise measurements of children’s PA might better identify the associations between the PA levels of parents and their children.

The aim of this study was to examine whether the time spent at different intensities of PA and the type of parental PA are associated with objectively measured daily PA of their 4 to 7-year old offspring. Since other studies previously found that the relationship between the PA of parents and their children depended on sex [30, 31], we specifically analysed the associations in child-parent pairs: mothers and daughters, mothers and sons, fathers and sons, and fathers and daughters. We hypothesized that children with more active parents are more physically active, compared to children with less active parents.

## Methods

### Participants

All children aged between 4 and 7 years, (mean age 6.1 ± 0.5 years) participating in the GECKO Drenthe birth cohort were included in the study. The GECKO Drenthe study is a population-based birth cohort studying early risk factors for overweight and obesity in children living in Drenthe, a northern province of the Netherlands. Parents and their babies born between 1 April 2006 and 1 April 2007 in Drenthe were recruited for the study. Details of the study design, recruitment and study procedures are described in detail elsewhere [32]. At baseline, the parents of 2997 children consented to participate, 2874 of whom actively participated in the study. Data were collected from the last trimester of pregnancy onwards by midwives and gynaecologists, and after birth during regular check-up visits to the Well Baby Clinics and municipal health services as part of the nationwide Youth Health Care programme which monitors the health, growth and development of children from birth to 18 years. Height and weight were measured by trained youth healthcare nurses at age six years during a regular check-up. The overweight and obesity of children was classified according to the cut-offs of Cole et al. [33]. Socioeconomic status (SES) was assessed by the education level of the parents (low/middle education or higher vocational education) and the highest household income, both registered during pregnancy. The height and weight of the parents were self-reported in questionnaires. Adult overweight was defined as BMI between 25 and 29.9 kg/m^2^ and obesity as ≥30 kg/m^2^. Written informed consent was obtained from parents and this study was approved by the Medical Ethics Committee of the University Medical Center Groningen in accordance to the 1975 Declaration of Helsinki, as amended in 1983.

### Physical activity

Between 2009 and 2013, families were contacted individually by research assistants to obtain data from parents and children simultaneously. PA in children was assessed using the ActiGraph GT3X (ActiGraph, Pensacola, FL) since the validity for measuring PA by questionnaire is low for children [34]. The ActiGraph is a reliable and valid device for measuring PA duration (minutes/day) at a certain intensity (sedentary behaviour (SB), light PA (LPA), moderate PA (MPA), vigorous PA (VPA) and moderate-to-vigorous PA (MVPA) in young children [35, 36]. The correlations between observed and ActiGraph intensity categorizations in young children ranged from 0.46 to 0.70 (*P* < 0.001) [35]. The ActiGraph device was worn by the child with an elastic belt. Parents were instructed to let their child wear the ActiGraph on the iliac crest on the right hip for four consecutive days, including at least one weekend day, during all waking hours except when bathing or swimming [37, 38]. To be included in the analysis for this study, the accelerometer had to be worn for at least 600 min/day for at least three days, regardless whether these were week or weekend days. Non-wearing time of the ActiGraph was defined as a minimum period of 90 min without any observed counts [39]. The cut-off points recommended by Butte et al. were used to calculate the time spent sedentary and in light PA (LPA) (240 counts per minute), LPA and moderate PA (MPA) (2120 counts per minute), and MPA and vigorous PA (VPA) (4450 counts per minute) [40]. The data collected were analysed in 15-s epochs [41]. Data were collected at a frequency of 30 Hz [42]. All the measurements for children with wearing times ≥840 min/day (14 h/day) were checked manually for sleeping time. Adherence to the Dutch PA guideline was defined in this study as ≥60 min of moderate to vigorous PA (MVPA) per day.

**Fig. 1:**
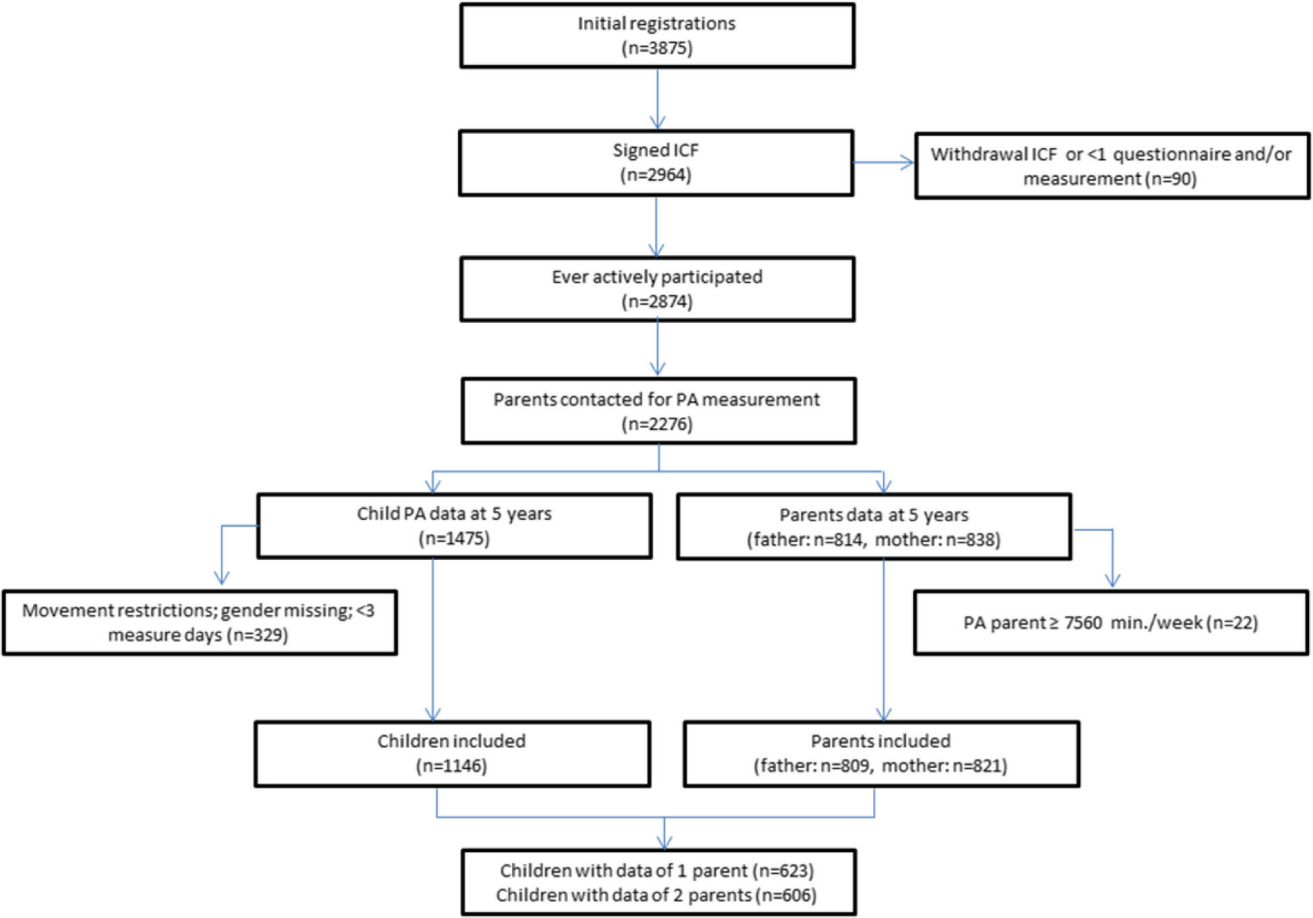
Flowchart of the participants. All participants were recruited from the GECKO Drenthe birth cohort (babies born between 1 April 2006 and 1 April 2007 in Drenthe, the Netherlands) and measured for PA between 2009 and 2012 when aged 4–7 years

Parental PA was assessed by the validated SQUASH (Short QUestionnaire to ASsess Health enhancing physical activity) [43] questionnaire, as self-reporting remains the most feasible and commonly used method for collecting data in large populations [44]. Overall reproducibility of the SQUASH is 0.58 (95%-CI 0.36–0.74). High-intensity activities are more reliable than low-intensity activities [43]. The SQUASH registers habitual physical activities and is pre-structured into four main domains: (a) commuting activities, (b) activities at work or school, (c) household activities, and (d) leisure time activities (including sports). Parents reported the time spent in each domain using three main queries: number of days in the preceding week, average time per day (in minutes) and intensity in three categories (light/slow, moderate or intense/fast). For PA at work, parents reported the number of hours in light and moderate PA (seated and standing work, such as office work) and the number of hours in vigorous PA (such as carrying heavy loads). The total PA in minutes per week was calculated and the outcomes were classified as time spent in light, moderate and vigorous PA, as well as the time spent in different types of PA, which were commuting, leisure time, sports, household tasks, and time spent in physical activities at work or school, according to Wendel-Vos et al. [43] The SQUASH questionnaire classifies a mixture of sedentary and light activities such as ‘office work’ under ‘light physical activity’ (LPA). Implausible values which were excluded were: 1) PA ≥ 18 h/day, 2) separate categories exceeding plausible values on that particular category, and 3) missing data for more than two questionnaire categories. Data for household activities were not analysed because they showed too much variation and were therefor considered less reliable [40]. Activities at work are conducted without children around and therefore no meaningful associations can be expected. The data cleaning was recorded and audited by a second investigator. If the investigators were unable to reach a consensus, a third researcher was consulted. Children were included in the present data analysis if valid PA data for the child and at least one parent were available (*N* = 623, Fig. 1). Participants were excluded from the analysis for various reasons: withdrawal of informed consent, completion of informed consent form but failure to participate in the study, failure to participate at follow up (no contact details for PA assessment due to moving to another province/country), unwillingness to participate in PA measurement, or logistical problems in the distribution of the questionnaires or ActiGraphs.

### Statistics

The data are presented as means with standard deviations, as rates in N and percentages, or if the data were skewed, as the median of the 25th and 75th percentiles. As most PA variables were more or less skewed, Spearman correlations were used to assess the associations between parental and child PA at different intensities. Since parental influence can be modified by education level, and both parental and child activities may be influenced by income level, education and income were investigated as potential modifiers in linear regression models. Dependent skewed variables were ln-transformed for linear regression. For reasons of clarity, only SQUASH results for sports, leisure time and active commuting are presented in Tables 2 and 3 alongside total PA (TPA), LPA, MPA, VPA and MVPA. The influence of gender was investigated by stratification. Finally, analysis was conducted to establish whether the children in families with two active parents were more active than children in families with inactive parents. For this, the parents were stratified into gender-specific tertiles based on MVPA and then regrouped into three categories: two active parents (both highest tertile), two inactive parents (both lowest tertile) and all other combinations. Statistical analyses were performed using IBM SPSS Statistics 22 for Windows (SPSS Inc., Chicago, IL). The graphs were prepared using GraphPad Prism 5.04 (GraphPad Software Inc., USA).

## Results

The parents of 2276 children were contacted and PA measurements of 1475 children, 838 mothers and 814 fathers were collected. As shown in Fig. 1, we obtained valid ActiGraph data from 623 children and PA data from at least one of their parents. Data from both parents were available for 606 of these children. The children were aged between 4.7 and 7.4 years (5.3–6.4, 5th–95th percentile). Age and BMI were comparable for boys and girls, and the boys were more active than the girls. The boys on average spent sixteen minutes per day more in MVPA (*p* < 0.001) and as a result more often satisfied the Dutch norm for healthy PA in children (Table 1). The total PA (TPA) data of the children included in the analyses were comparable to data of the children who were excluded for lack of parental data. The TPA data for the parents with child PA data were also comparable to the TPA for the parents without valid child PA data (data not shown).

**Table 1:** Descriptive characteristics of children and parents in the GECKO Drenthe cohort

Child factors	N	Girls	N	Boys	*P*-value
Age at PA measurement (years)	299	6.1 ± 0.5	324	6.1 ± 0.5	0.93
Ethnicity (%)	282		304		0.32
Dutch		94.7		96.4	
Non-Dutch		5.3		3.6	
Highest household income, N (%)	267		279		0.22
≤ EUR1150		11 (4)		6 (2)	
EUR1151–3050		179 (67)		173 (62)	
EUR3051–3500		51 (19)		69 (25)	
≥ EUR3501		26 (10)		31 (11)	
BMI (kg/m^2^)	262	16.0 ± 1.5	277	16.0 ± 1.2	0.57
Physical activity (PA)	299		324		
Total PA (counts/minute, cpm)		764 ± 197		839 ± 241^a^	< 0.001
Sedentary (hrs/day)		6.41 ± 0.92		6.27 ± 0.96	0.053
Light PA (hrs/day)		4.28 ± 0.61		4.31 ± 0.62	0.58
Moderate PA (min./day)		40 (31; 48)		48 (40; 61)^a^	< 0.001
Vigorous PA (min./day)		16 (11; 24)		20 (14; 28)	< 0.001
Moderate-to-vigorous PA (min./day)		55 (43; 72)		71 (54; 90)	< 0.001
Adherent to PA guideline, N (%)		132 (44)		215 (66)	< 0.001
Parent factors	N	Mothers (*n* = 621)	N	Fathers (*n* = 608)	
Age (years)	620	37.1 ± 4.4	579	40.0 ± 5.0	
BMI at PA measurement (kg/m^2^)	417	24.6 ± 4.0	372	25.4 ± 3.1	
Overweight and obesity, N (%)	417	164 (39.3)	372	178 (48.0)	
Education level (%)	595		578		
Low/middle		348 (58.5)		378 (65.4)	
High (higher vocational)		247 (41.5)		200 (34.6)	
Physical activity (PA)	621		608		
Total PA (hrs/day)		8.0 (6.3–10.3)		7.9 (6.6–9.4)	
Light PA (hrs/day)		6.5 (4.7–8.4)		6.0 (2.7–7.4)	
Moderate PA (hrs/day)		1.14 (0.57–2.50)		0.93 (0.29–4.14)	
Vigorous PA (hrs/day)		0.00 (0.00–0.26)		0.14 (0.00–0.43)	
Moderate-to-vigorous VPA (hrs/day)		1.29 (0.71–2.77)		1.32 (0.57–4.56)	
Sports (hrs/day)		0.14 (0.00–0.36)		0.14 (0.00–0.43)	
Leisure time PA (hrs/day)		0.79 (0.43–1.38)		0.89 (0.50–1.53)	
Housework (hrs/day)		3.57 (2.29–6.00)		1.00 (0.29–2.00)	
Active commuting (hrs/day)		0.00 (0.00–0.10)		0.00 (0.00–0.07)	
Active work (hrs/day)		3.43 (2.14–3.86)		5.71 (4.57–6.07)	

All the participants were recruited from the GECKO Drenthe birth cohort (babies born between 1 April 2006 and 1 April 2007 in Drenthe, the Netherlands). The data are presented as means ± standard deviations or as medians of the 25th and 75th percentiles or as percentages

Table 2 shows the associations between parental and child PA. Generally, the parents’ total PA was not associated with the PA levels of their offspring. No clear associations could be found between the intensities of the parent’s PA and their children’s. Only more time in light PA for fathers was associated with lover levels of MPA in children. With respect to the type of activities, we found that the children of mothers with higher leisure time PA had higher MVPA levels and less sedentary time. This sedentary behaviour in children was also related to more sports activity in mothers, but also to more active commuting time in mothers.

**Table 2:** Spearman’s correlations between the PA of the mothers and fathers and their children

	All children					
	TPA	Sedentary behaviour	LPA	MPA	VPA	MVPA
Physical activity (PA) Mother						
Total PA	0.018	−0.013	0.023	0.016	0.030	0.020
Light PA	0.003	0.024	0.004	−0.023	0.003	−0.020
Moderate PA	0.000	−0.070	0.042	0.041	0.007	0.035
Vigorous PA	0.047	−0.038	0.004	0.072	0.068	0.072
Moderate-to-vigorous PA	0.019	−0.076	0.044	0.062	0.020	0.052
Sports	0.040	−0.083*	0.070	0.058	0.066	0.064
Leisure time PA	0.052	−0.082*	0.067	0.073	0.065	0.079*
Active commuting	−0.050	0.085*	−0.032	− 0.048	− 0.011	−0.030
Physical activity (PA) Father						
Total PA	0.010	0.013	0.078	−0.046	0.010	−0.022
Light PA	−0.032	0.051	0.025	−0.098*	−0.031	− 0.079
Moderate PA	0.027	−0.021	0.011	0.030	0.011	0.030
Vigorous PA	0.024	−0.014	0.038	0.066	0.056	0.064
Moderate-to-vigorous PA	0.041	−0.028	0.019	0.056	0.035	0.056
Sports	−0.010	0.000	0.000	0.041	0.049	0.040
Leisure time PA	−0.002	− 0.039	0.080	− 0.021	0.015	0.001
Active commuting	−0.034	0.064	−0.024	−0.040	− 0.038	−0.040

All the participants were recruited from the GECKO Drenthe birth cohort (babies born between 1 April 2006 and 1 April 2007 in Drenthe, the Netherlands) and measured for PA between 2009 and 2012 when aged 4–7 years

*p < 0.05, values indicate Spearman’s rho

Table 3 presents the specific parent-child pair correlations. No associations were found regarding total PA. Regarding the time spent in PA of different intensities, higher levels of MPA, VPA or MVPA in parents were generally related to higher levels of MVPA in sons or daughters. More specifically, higher VPA in both mothers and fathers was related to higher MVPA in daughters, whereas for sons, only paternal MPA and MVPA were significantly associated with MVPA in sons. A higher level of VPA in mothers was also expressed in the association between more time in sports and leisure time PA in mothers and more MVPA in daughters. In contrast, higher levels of light PA in fathers correlated with lower levels of MVPA in sons, and reciprocally also with more time in sedentary behaviours in sons, but not in daughters. Comparing families with two active parents, families with one active parent and families with two inactive parents, no other associations were found for PA in children than those already described (data not shown).

**Table 3:** Spearman’s correlations between the PA of the mothers and fathers stratified for sons and daughters

	Sons		Daughters	
	Sedentary	MVPA	Sedentary	MVPA
Physical activity (PA) Mother				
Total PA	−0.062	0.000	0.040	0.078
Light PA	−0.027	−0.038	0.076	0.035
Moderate PA	−0.055	0.034	−0.085	0.057
Vigorous PA	0.026	0.007	−0.051	0.159*
Moderate-to-vigorous PA	−0.070	0.047	−0.080	0.078
Sports	−0.072	−0.003	− 0.091	0.133*
Leisure time PA	−0.080	0.059	−0.084	0.127*
Active commuting	0.037	−0.054	0.146*	−0.016
Physical activity (PA) Father				
Total PA	0.041	−0.092	−0.014	0.075
Light PA	0.122*	−0.228**	−0.019	0.072
Moderate PA	−0.079	0.147*	0.038	−0.067
Vigorous PA	0.049	−0.027	−0.083	0.141*
Moderate-to-vigorous PA	−0.061	0.132*	0.002	−0.003
Sports	0.069	−0.046	−0.073	0.103
Leisure time PA	−0.036	−0.002	− 0.053	0.021
Active commuting	0.087	−0.080	0.045	−0.026

All participants were recruited from the GECKO Drenthe birth cohort (babies born between 1 April 2006 and 1 April 2007 in Drenthe, the Netherlands) and measured for PA between 2009 and 2012 when aged 4–7 years

*p < 0.05, **p < 0.01, values indicate Spearman’s rho

We hypothesized that more active fathers would have more active children, and thus, that fathers with more time spent in light PA would have be positively association with more time spent in higher intensities of activity in children. A positive association between light PA of the father and sedentary time of the son was an unexpected finding. We subjected this latter finding to further study, and especially aimed to understand the nature of the light PA of the father. Part of the LPA in the SQUASH questionnaire come from work-related activities, including office work (sitting/standing work, walking now and then, or walking with light carrying activities). Since this type of work may be related to a higher socioeconomic position of the family, we studied the effects of family income and parental education level on the associations between parental PA and child PA. Parents’ income and education levels were found not to influence the association between parental PA and child PA in general (data not shown), though the association between paternal LPA and a son’s sedentary behaviour could be fully explained by the father’s education level (Table 4).

**Table 4:** The association between paternal LPA and sedentary time in sons is explained by paternal education level

	Std. B	β	95% CI (β)	*P*-value
Crude model for paternal LPA and sedentary time of sons				
Paternal LPA (min/week)	0.144	0.164	0.027–0.301	0.019
Crude model for paternal education level and sedentary time of sons				
Paternal education level	0.121	0.245	0.017–0.473	0.035
Adjusted models				
Model 1				
Paternal LPA	0.100	0.113	−0.030–0.256	0.119
Paternal education level	0.141	0.280	0.030–0.531	0.029
Model 2				
Paternal LPA (min/week)	0.100	0.114	−0.030–0.257	0.119
Paternal education level	0.137	0.272	0.0001–0.544	0.050
Family income	0.011	0.014	0.160–0.188	0.873

Std. B, standardized β coefficient; 95% CI (β), 95% confidence interval of the β coefficient. Model 1 presents the mutually adjusted model for explaining sedentary time in sons using both LPA and the father’s education level. Model 2 presents further adjustment of model 1 by family income. Paternal LPA was ln-transformed to obtain a normal distribution of the residuals. The outcome ‘sedentary time in sons’ is used in hours/day and was normally distributed. For interpretation, a β of 0.245 for paternal education level means fifteen more minutes per day (0.245 h) of sedentary behaviour for the sons of high and low educated fathers

No direct effect of income was found on the association between paternal LPA and sedentary time in sons (model 2, Table 4). Still, fathers with a high level of LPA spent more time in occupational PA (5.3 against 6.1 h for the lowest vs. highest LPA tertiles, *p* < 0.001), which was mostly classified as light activity (office work and intermittent sedentary work) and they had a higher income (15% against 41% highest income for the lowest vs. highest LPA tertiles, *p* = 0.009 for Chi^2^ test).

## Discussion

This study found that mothers who spent more time in VPA and more time in sports and leisure time PA had daughters who spent more time in MVPA, whereas fathers with higher levels of MPA and MVPA had sons with higher levels of MVPA. These findings support the hypothesis that higher levels of more intense PA in parents are associated with higher levels of more intense PA in children, and support a modelling role of parents in the PA levels of their children that is sex-specific. Furthermore, higher levels of active commuting in mothers were associated with more sedentary time in daughters, and higher levels of LPA in fathers were associated with more sedentary time in sons. This latter association could be fully explained by the education level of the fathers, suggesting that children from families where the parents have sedentary jobs seem to adopt more sedentary behaviour, as well.

Although many studies have been published on the association between parental PA and child PA, few used objective measurements (e.g. accelerometry) to assess activity levels in preschoolers and distinguished the gender-specific relationships between parental PA and child PA as well. Some studies used accelerometry only in children [45–48] and two studies also used accelerometry in parents [49, 50]. Most previous studies support the idea that the PA level of parents is associated with the PA of preschoolers [45, 49–51]. Two studies showed no clear association with parental PA, which could be explained by their use of self-reported PA rather than objectively measured PA, or differences in socioeconomic status [46, 47]. Additionally, in 431 children of 10–12 years old, Jago et al. did not find an association between PA in parents and children, despite the use of accelerometry for both [52]. The contradictory results in that study could be explained by the children’s higher age, since research focusing on age-related social influences found that associations with PA in adolescents shift from parents to peers [53]. A more recent study by Jago et al. in 1267 five- and six-year-olds showed a weak association between the MVPA of children and their parents [51]. Both the children’s and the parents’ PA in that study were measured using accelerometry. Other PA intensities were not reported.

Overall, we found differences between the role of the mothers’ and the fathers’ PA on their children’s PA. Considering boys and girls together, maternal PA was more often and more significantly associated with PA and sedentary behaviour, compared to paternal PA. Previously, Taylor et al. showed that paternal PA was more predictive of child activity than maternal PA [49]. Sallis et al. only studied the role of maternal PA on children’s PA, and not paternal PA, and found no association [46]. Our previous study of the GECKO Drenthe cohort at toddler age (3–4 years) found that maternal active commuting was associated with lower BMI and higher levels of light physical activity in children [54]. This could be explained by the mothers’ active participation in daily PA with their children, contributing to healthier BMI in their children.

Active fathers had active sons. This positive correlation between fathers’ and sons’ PA was observed previously [51] and was also found in a review of 150 mostly cross-sectional studies with subjective measurement of PA, i.e. questionnaires or observation [55]. Recently, Vollmer et al. also found a positive correlation between the VPA of 150 fathers and the VPA of their 3 to 5-year-old children in one-on-one interviews [56]. In addition, and more unexpectedly, we found that paternal LPA had a significant positive correlation with sedentary time and an inverse association with the MVPA of boys. Considering the determinants of paternal LPA in our study, this included time spent at work (*r* = 0.23, *P* < .001). Accordingly, doing seated office work was counted as time spent in LPA, while it was actually time spent in sedentary time. Here, low paternal LPA was correlated with lower offspring sedentary time. Father-child associations using objectively measured sedentary time show mixed findings [52, 57, 58]. Fathers with lower LPA in our study were younger, had lower incomes and education levels, did less housework and had higher BMI (data not presented). Although children with higher income parents are presumed to have healthier lifestyles [59], fathers and sons with lower SES may not necessarily be less active because SES is unrelated to PA in pre-schoolers and school-aged children [60] but is related to sedentary time [52]. This difference in the attribution of determinants for PA and sedentary behaviour could explain why the association between lower LPA in fathers and lower levels of sedentary time in boys ceased to be significant when controlling for education level. In general, it is important that determinants for PA and sedentary behaviour are different and therefore the mechanism by which higher and lower levels of PA and sedentary behaviour are determined are different. The fathers in our study with low LPA may have had lower SES, and also different interactions with their boys than the older, lower BMI, more highly educated and higher income fathers. The father’s type of work, the time available for children, the money available for sports or video gaming equipment and television viewing, the neighbourhood where the child grows up, and the father’s knowledge of health behaviour are all factors which could affect the association between the activity levels of fathers and boys.

The representativeness of the study population, its large sample size and the objective measurement of PA in children are all important strengths of this study. This study included 623 parent/child combinations, which is more than other studies of young children (*n* = 33 to *n* = 347) [45–47, 49, 50]. The selection bias was weak, since parents were not recruited specifically for a PA study, but recruited from among the participants in the birth cohort [32]. In addition, no differences were found between the PA levels of children for whom parental data was or was not recorded. The number of children who met the Dutch activity norm for a healthy lifestyle was 44% for girls and 66% for boys, which corresponds reasonably well with the national average of 50% in children aged 4–11 years between 2006 and 2012 [61]. The PA in children was objectively measured, which is more accurate than PA measured by questionnaires [19].

## Conclusion

Mothers who spent more time in vigorous PA, for instance in sports or leisure time activities, had more active daughters. Fathers who spent more time in moderate intensity PA had more active sons. The intensity of the parents’ physical activity seemed to be particularly related to the time spent in moderate intensity activity in children, but not to sedentary time. Sedentary activities of children may be dependent on other family-related factors such as the parents’ occupational characteristics. To encourage PA in young children, interventions could focus on the PA of parents, taking into account that especially fathers influence their sons and mothers their daughters. Further research is warranted into the parents’ occupational factors, as children seem to adopt more sedentary habits in families where the parents have sedentary work.

## Abbreviations

BMI: Body Mass Index
GECKO: Groningen Expert Center for Kids with Obesity
IBM: International Business Machines Corporation
LPA: Light Physical Activity
MVPA: Moderate to Vigorous Physical Activity
PA: Physical Activity
SES: Socioeconomic Status
SPSS: Statistical Packages in Social Science
SQUASH: Short QUestionnaire to ASsess Health enhancing physical activity
VPA: Vigorous Physical Activity
